# Comment on “The GalNAc-T Activation Pathway (GALA) is not a general mechanism for regulating mucin-type O-glycosylation”

**DOI:** 10.1371/journal.pone.0180005

**Published:** 2017-07-18

**Authors:** Frederic Bard, Joanne Chia

**Affiliations:** Institute of Molecular and Cell Biology, Proteos, Singapore, Singapore, and Department of Biochemistry, National University of Singapore, Singapore, Singapore; Institut Curie, FRANCE

In the PLOS ONE article “The GalNAc-T Activation Pathway (GALA) is not a general mechanism for regulating mucin-type O-glycosylation”, Tabak and colleagues argue that they cannot reproduce part of the results we published in 2010 [1]. Specifically, the fact that EGF and PDGF growth factors stimulation of HeLa cells results in a relocation of the O-glycosylation initiation enzymes polypeptide N-acetylgalactosaminyltransferase (GALNTs) from the Golgi apparatus to the endoplasmic reticulum (ER).

As O-GalNAc glycosylation concerns a large score of cell surface and secreted proteins and has been shown to affect biological functions in many cases, the question of the regulation of activity of GALNTs enzymes is of significant importance. One aspect of O-glycosylation regulation is the differential gene expression of GALNTs family members. Others have shown the differential glycosylation repertoire of GALNTs and the differential gene expression of some GALNTs family members depending on tissue or developmental stage [2,3]. We were the first to propose that signalling pathways and in particular, the tyrosine kinase Src regulate the GALNTs sub-cellular localisation and can induce a relocation from the Golgi apparatus to the ER. We found relocation to occur for the most abundantly expressed enzymes of the family (GALNT1, -T2, -T3) as well as for GALNT4 and -T6. We showed that this Golgi to ER relocation results in an upregulation of GALNTs activity, hence the name GALNTs Activation (GALA) pathway.

GALNTs catalyse the formation of the Tn antigen, formed by a GalNAc residue alpha-linked to a Serine or Threonine residue on a polypeptide. These GalNAc residues are usually biosynthetic intermediates for more complex glycans [4]. Tn can be recognised by lectins such as *Vicia villosa* lectin (VVL) and *Helix pomatia* lectin (HPL). GALA results in an increase in cellular Tn staining, because more Tn is formed and also because it is not capped in the ER as it is in the Golgi [5].

Over the last months, we have revisited the question of stimulation of GALNTs relocation by growth factors. We have been able to reproduce several of our 2010 results and have identified a couple of potential explanations for the discrepancy with Dr. Tabak’s group results.

1. Tabak’s group chose to quantify directly the change in GALNTs location at the Golgi by measuring Manders’ correlation coefficient between GALNTs and TGN and ER markers (TGN46 and Calnexin). They used a full volume confocal sectioning (30 slices) with a pinhole set at 0.7 Airy Unit. While this is a good approach to detect colocalisation in vesicular structures where the signal is concentrated, it is challenging with weaker, more diffuse signals. Indeed, 3D confocal sectioning tends to bleach weak signals due to extensive illumination and a small pinhole aperture tends to reduce the detection limit of the instrument. The ER is more than 10x larger than the Golgi and is distributed in the whole cell volume in sheets and tubules. Furthermore, GALNTs are not very abundant proteins, so they become highly diluted after relocation and are difficult to detect in the ER, even when the relocalisation is extensive. In these conditions, it is unlikely that the imaging experiment would be able to pick up the GALNTs ER signal. For illustration purposes, we show an experiment using ERK8 depletion as a strong inducer of relocation: compared with the Golgi signal, it is harder to detect GALNTs in the ER (Fig 1).
2. In Fig 3 of their paper, Herbomel et al. used ERK1/2 activation to validate the bioactivity of their growth factors. However, this is not necessarily a very good reflection of the tyrosine kinase cascade activation leading to Src activation at the Golgi. Indeed, ERK activation functions as on/off switch thanks to a feed-forward loop; even a small amount of EGF can lead to full ERK1/2 activation [6]. The pathway is then inactivated by phosphatases. By contrast, GALA functions not like an on/off switch but like a rheostat; variable amounts of enzymes can be relocated. The inactivation of GALA relies on the normal flow of glycosylation enzymes back to the Golgi; GALNTs do not appear to be retained in the ER [7]. Thus, ER localisation of GALNTs requires an activation of retrograde Golgi-to-ER traffic and is constantly and dynamically counter-balanced by anterograde ER-to-Golgi traffic [7]. Thus, a moderate activation of GALA results in an increased cycling of the enzymes through the ER, with an increase in Tn levels and ER resident protein glycosylation but without a large net displacement of the enzymes. We found that EGF and PDGF stimulation in HeLa cells produce a moderate activation of tyrosine kinase cascades and the GALA pathway. We also observed that this stimulation is also a bit finicky and variable, requiring extensive washing of the fetal bovine serum in culture medium and starvation.

**Fig 1:**
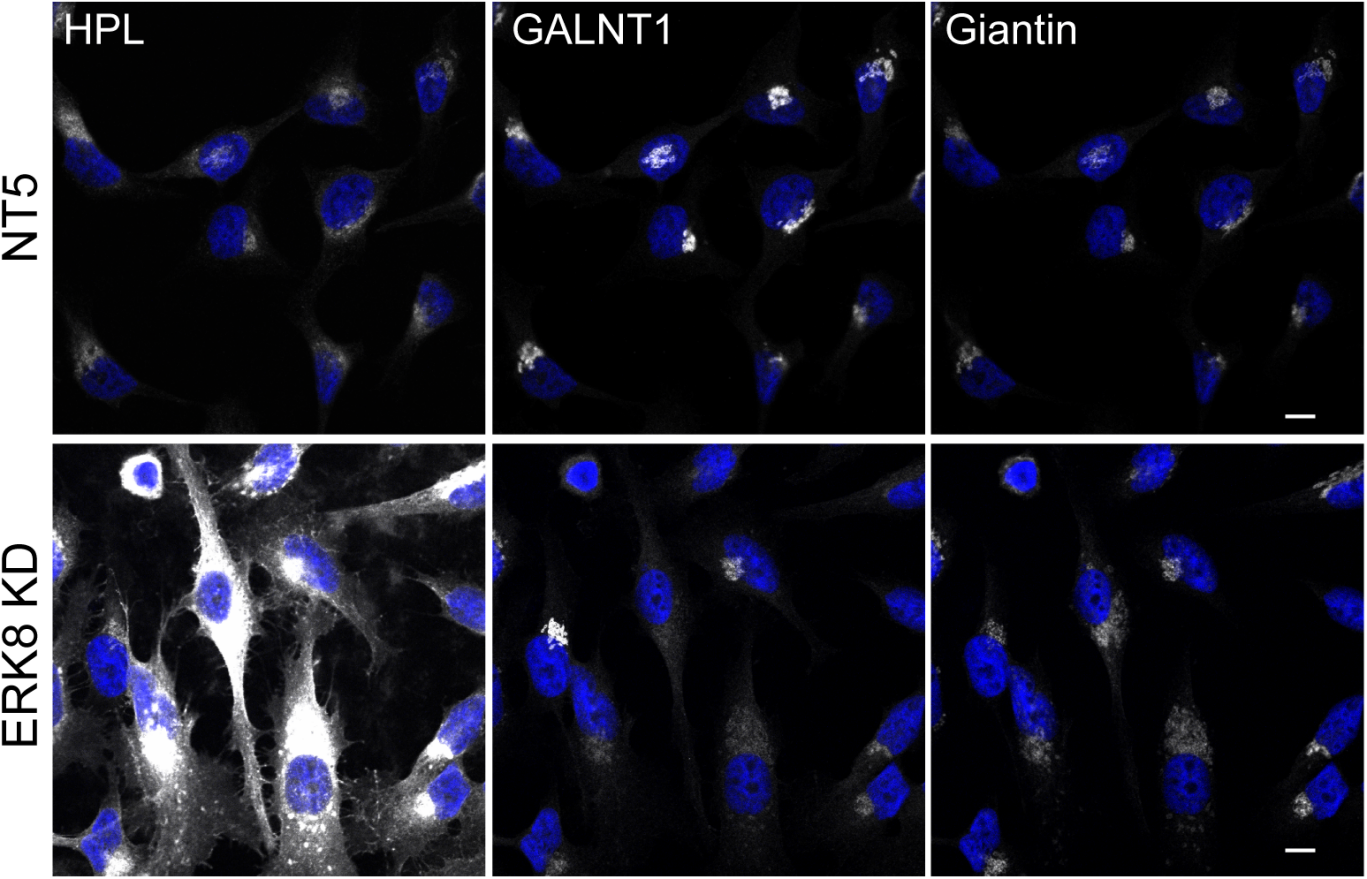
GALNT1 signal in the ER.

For people interested in exploring GALNTs relocation and its physiological effects, we would recommend using first, ERK8 depletion to induce a marked GALNTs relocation in HeLa cells [7]. It is also possible to use an active form of Src [1]. Expression of strongly active Src comes with an additional caveat as it tends to also fragment the Golgi apparatus in HeLa cells. However, this Src induced fragmentation is much more limited in fibroblastic cell lines such as WI-38 and MEFs [1]. We also recommend using Tn staining and quantifying total cell intensity. Because Tn is the result of an enzymatic reaction and found on many carrier proteins, its detection is usually easier than GALNTs detection. Tn staining intensity with fluorescently labelled lectins can be reliably quantified, with a relatively large dynamic range.

Ultimately, the best context to study the effects of GALA may be in cancer cells *in situ*. Indeed, in a large majority of the breast/mammary and liver tumors we tested, the pathway is strongly activated with several fold increase in Tn levels [8]. Intriguingly, the levels of Tn and GALA are not very strong in the cancer cell lines we tested [8]. A down-regulation of the pathway seems, therefore, to occur during the establishment of these lines, suggesting that the drivers of the pathway *in vivo* are lost. What is the nature of these drivers remains a fascinating question for the future.

While we regret that we were not able to resolve the discrepancies in our exchanges with Dr. Tabak, we are thankful to him for keeping us abreast of their results and progress with the manuscript.

In this experiment, GALNT1 relocation was induced by ERK8 depletion (“ERK8 KD”) as described in [7]. Imaging was performed at 40X on a confocal microscope under immersol oil (Zeiss inverted confocal LSM700). Identical imaging conditions were used for both set of conditions, non-targeting siRNA (NT5) and ERK8 KD. Scale bar, 10μm.

## References

[1] Gill DJ, Chia J, Senewiratne J, Bard F. Regulation of O-glycosylation through Golgi-to-ER relocation of initiation enzymes. J Cell Biol. 2010;189: 843–858. doi: 10.1083/jcb.201003055 20498016 10.1083/jcb.201003055 PMC2878949

[2] Tian E, Ten Hagen KG. Expression of the UDP-GalNAc: polypeptide N-acetylgalactosaminyltransferase family is spatially and temporally regulated during Drosophila development. Glycobiology. 2006;16: 83–95. doi: 10.1093/glycob/cwj051 16251381 10.1093/glycob/cwj051

[3] Bennett EP, Mandel U, Clausen H, Gerken TA, Fritz TA, Tabak LA. Control of mucin-type O-glycosylation: a classification of the polypeptide GalNAc-transferase gene family. Glycobiology. 2012;22: 736–756. doi: 10.1093/glycob/cwr182 22183981 10.1093/glycob/cwr182 PMC3409716

[4] Gill DJ, Clausen H, Bard F. Location, location, location: new insights into O-GalNAc protein glycosylation. Trends Cell Biol. 2011;21: 149–158. doi: 10.1016/j.tcb.2010.11.004 21145746 10.1016/j.tcb.2010.11.004

[5] Bard F, Chia J. Cracking the Glycome Encoder: Signaling, Trafficking, and Glycosylation. Trends Cell Biol. 2016;26: 379–388. doi: 10.1016/j.tcb.2015.12.004 26832820 10.1016/j.tcb.2015.12.004

[6] Ryu H, Chung M, Dobrzyński M, Fey D, Blum Y, Lee SS, et al Frequency modulation of ERK activation dynamics rewires cell fate. Mol Syst Biol. 2015;11: 838 doi: 10.15252/msb.20156458 26613961 10.15252/msb.20156458 PMC4670727

[7] Chia J, Tham KM, Gill DJ, Bard-Chapeau EA, Bard FA. ERK8 is a negative regulator of O-GalNAc glycosylation and cell migration. Elife. 2014;3: e01828 doi: 10.7554/eLife.01828 24618899 10.7554/eLife.01828 PMC3945522

[8] Gill DJ, Tham KM, Chia J, Wang SC, Steentoft C, Clausen H, et al Initiation of GalNAc-type O-glycosylation in the endoplasmic reticulum promotes cancer cell invasiveness. Proc Natl Acad Sci U S A. 2013;110: E3152–61. doi: 10.1073/pnas.1305269110 23912186 10.1073/pnas.1305269110 PMC3752262

